# Flying to the moon: Impactful accounts of triatomines invasion from the 2nd to the 13th floor of an urban residential building in the municipality of Rio Branco, Acre, Brazil

**DOI:** 10.1590/0037-8682-0122-2024

**Published:** 2024-09-02

**Authors:** Manoella da Silva Moura, Luciana Braga da Silva, Fernanda Portela Madeira, Francisco Warcron Oliveira das Neves, André Luiz Rodrigues Menezes, João Aristeu da Rosa, Jader de Oliveira, Luís Marcelo Aranha Camargo, Mariane Albuquerque Lima Ribeiro, Dionatas Ulises de Oliveira Meneguetti

**Affiliations:** 1 Universidade Federal do Acre, Programa de Pós-Graduação em Ciências da Saúde na Amazônia Ocidental, Rio Branco, AC, Brasil.; 2 Universidade Federal do Acre, Centro Multidisciplinar, Campus Floresta, Cruzeiro do Sul, AC, Brasil.; 3 Programa de Pós-Graduação em Ciência, Tecnologia e Inovação para Amazônia, Universidade Federal do Acre, Rio Branco, AC, Brasil.; 4 Instituto Federal de Educação, Ciência e Tecnologia de Rondônia, Guajará Mirin, RO, Brasil.; 5 Faculdade de Ciências Farmacêuticas da Universidade Estadual Paulista "Júlio de Mesquita Filho", Araraquara, SP, Brasil.; 6 Laboratório de Entomologia em Saúde Pública, Faculdade de Saúde Pública, Universidade de São Paulo, São Paulo, SP, Brasil.; 7 Instituto de Ciências Biomédicas 5, Universidade de São Paulo, Monte Negro, RO, Brasil.; 8 Universidade Federal do Acre, Centro de Ciências da Saúde e do Desporto, Rio Branco, AC, Brasil.; 9 Universidade Federal do Acre, Colégio Aplicação, Rio Branco, AC, Brasil.

**Keywords:** Chagas disease, Vectors, Amazon

## Abstract

**Background:**

Vectorial transmission through hematophagous triatomine insects remains the primary mode of Chagas Disease contagion. These insects have become increasingly common in urban environments. Therefore, this study aimed to report an encounter of triatomines with trypanosomatid infection in a vertical residential condominium in Rio Branco, the capital of the state of Acre, in the western Brazilian Amazon.

**Methods:**

Triatomines were collected from residents and sent to the municipality's Entomological Surveillance sector. Trypanosomatid positivity was evaluated using optical microscopy, followed by species and genotype identification using molecular biology techniques.

**Results:**

Twenty-five adult triatomine specimens were collected from two of three condominium buildings invading apartments from the 2nd to 13th floors. Six specimens were identified as *Rhodnius* sp. and 19 as *R. montenegrensis*. Among these, molecular tests were conducted on seven specimens, with five testing positive for *Trypanosoma cruzi*, all belonging to genotype TcI.

**Conclusions:**

These findings underscore the need for further studies to better understand the invasive capacity of these insects in these environments and the mechanisms involved in this process.

## INTRODUCTION

Chagas disease (CD), or American trypanosomiasis, is considered one of the most important public health issues in Latin America, especially in Brazil, a country estimated to have one million individuals infected with *Trypanosoma cruzi*, the etiological agent of this disease^1^ ^,^ ^2^. This parasite is genetically diverse, grouped into six genotypes, known as Discrete Typing Units (DTUs). Each of these *T. cruzi* strains may lead to different clinical manifestations depending on the location and the infected host^3^ ^,^ ^4^.

In Brazil, the Amazon region stands out in cases of this disease, where the predominant transmission routes are oral, through the ingestion of food contaminated with *T. cruzi*, and vector-borne, through the feces of infected hematophagous triatomine bugs^1^ ^,^ ^5^ ^,^ ^6^. Triatomine bugs belong to the family Reduviidae and subfamily Triatominae, with the latter currently comprising 18 genera and 159 described species, all of which (with the exception of three fossils) have the potential to transmitting *T. cruzi* ^7^ ^-^ ^9^. 

 In the Brazilian Amazon alone, 8 genera and 22 species of triatomine bugs have been recorded, with species of the genera *Rhodnius* and *Panstrongylus* being of greater epidemiological importance in the region^8^ ^,^ ^10^. The distribution of triatomine species and the environmental degradation associated with the migratory flow that the Amazon Basin has been experiencing over the years have intensified the movement of these insects into areas increasingly closer to human contact, with growing reports of intradomiciliary and peridomiciliary invasions^11^ ^-^ ^13^. Modification of the forest landscape into a more urbanized landscape influences these invasions, especially species of the genus *Rhodnius*, which have already demonstrated the ability to adapt to deforested areas with palm trees near residences^14^.

This dispersal of triatomine bugs is epidemiologically significant in the transmission of Chagas disease and can occur in two ways: passively, when they are carried through objects or some host animal, and actively, through the terrestrial movement of nymphs and adults, mainly by flight of adults^15^ ^,^ ^16^. However, their ability to fly remains poorly understood. Some researchers have suggested in their studies that environmental factors and nutritional status influence triatomine dispersal^17^ ^,^ ^18^. A laboratory experiment concluded that *R. brethesi* initiated take-off approximately 15 days after the last blood meal, suggesting that fasting boosted its flying ability^18^. However, it has also been observed that despite favorable conditions, some species of triatomine bugs do not engage in flights^18^.

Nevertheless, this dispersal, albeit less discussed, is important for maintaining the cycle of Chagas disease transmission, considering that many triatomine bugs are naturally infected by *T. cruzi* and show greater vectorial infection abundance in deforested areas than in preserved forest areas^19^ ^-^ ^20^. In line with this, the present study aimed to record the occurrence of triatomine bugs in apartments and their infection by trypanosomatids in the municipality of Rio Branco, Acre, Brazil, and, to the best of our knowledge, the presence of triatomine bugs on the 13th floor of a residential building.

## METHODS

**FIGURE 1:**
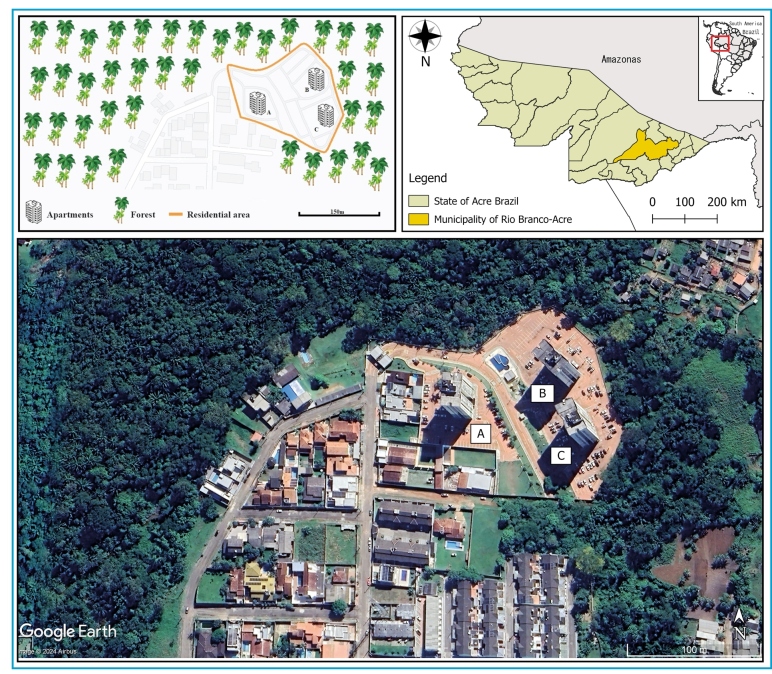
Characteristics of the residential condominium with triatomine invasion, Rio Branco, Acre, Brazil, 2023. A, B, and C represent the buildings housing the apartments.

Triatomine bugs were collected in the municipality of Rio Branco, Acre, Brazil, from July 2022 to June 2023 by direct capture by apartment residents. The specimens were delivered to the Entomological Surveillance Sector of Rio Branco, Acre, Brazil. The condominium comprises three buildings A, B and C (A: 9°57'26.58"S, 67°50'40.09"W; B: 9°57'25.89"S, 67°50'40.06"W; and C: 9°57'27.35"S, 67°50'39.73"W), each with 1 ground floor plus 16 floors, each floor 2.8 m high, so the total height is 47.6 m (Figure 1). Both buildings were located near forest fragments filled with palm trees. The main balcony of Building A is at least 100 m from Buildings B and C, which are 20 m apart.

The insects were sent in thermal boxes, kept at room temperature, to the Laboratory of Tropical Medicine (LABMEDT) at the Federal University of Acre (UFAC), where species identification was performed considering the morphological characteristics described by Lent and Wygodzinsky^21^ and Rosa et al.^22^ Some of the insects found in the apartments were not captured by the residents because of fear of contact with the insect; however, as the residents took photographic records, these reports were included in the study.

From the captured triatomine bugs, the contents of the rectal ampoule were extracted, diluted in saline solution (0.9%), and evaluated using optical microscopy (400x × magnification) to verify the presence of trypanosomatids. Trypanosomatid DNA was extracted from the rectal ampoules for subsequent molecular species and genotype identification.

DNA extraction followed the protocol described by Adams et al.^23^, using Digsol solution (50 mM Tris, 20 mM EDTA, 117 mM NaCl, and 1% SDS) to digest the feces and digestive tract overnight at 37°C. Subsequently, it was precipitated with ammonium acetate solution, centrifuged, and washed with 99.8% and 70% ethanol. The tubes were dried, with DNA resuspended in 40 μl of TE buffer (Tris-EDTA), and stored at -20°C.

After DNA extraction, the concentration and purity of each extracted sample were verified using NanoDrop™️. To confirm the trypanosomatid species, the fluorescent fragment length coding (FFLB) method was employed, allowing for the simultaneous identification of trypanosome species or genotypes, including mixed infections^24^ ^,^ ^25^. The technique was based on the amplification of four variable regions of the 18S and 28Sα rRNA genes to identify trypanosome species based on the observed polymorphisms between them^24^ ^,^ ^26^. DNA samples from triatomine bugs were subjected to four PCR using fluorescent primers^24^ ^,^ ^25^ ^,^ ^27^ ^,^ ^28^ described by Hamilton et al.^27^.

## RESULTS

A total of 25 adult triatomine bugs were found on different floors of buildings B (two specimens) and C (23 specimens) (Figure 1), which were closest to the forest fragment, approximately 20m away. The triatomines were collected from the 2nd (5.6 to 8.4m high) to the 13th floor (36.4 to 39.2m high). The floors with the highest occurrence were the 7th with nine specimens and the 2nd with six specimens, both in Building C, in a single apartment per floor. The remaining triatomines were distributed across the different apartments.

**FIGURE 2:**
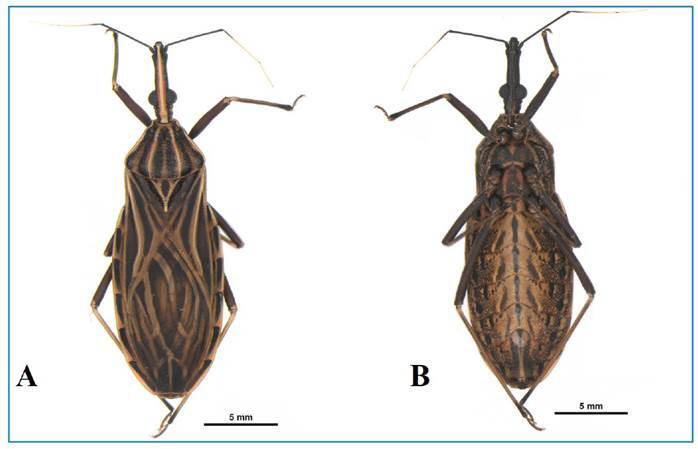
*Rhodnius montenegrensis* captured in the residential condominium in Rio Branco, Acre, Brazil, 2023. Caption: Female: **A)** Dorsal view. **B)** Ventral view.

**FIGURE 3:**
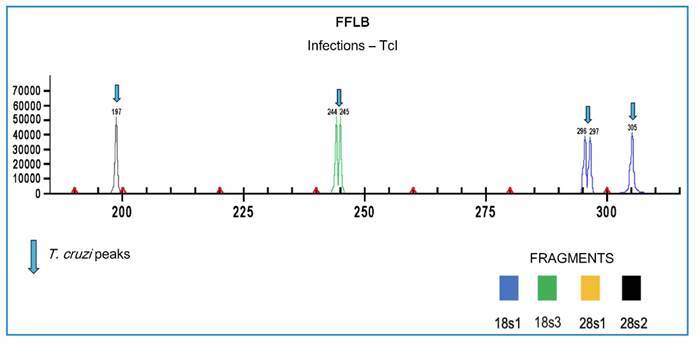
FFLB profiles obtained with DNA samples from *R. montenegrensis*. The image shows different peaks that form the specific profile of *T. cruzi* (TcI).

Of the 25 triatomines found, six were only recorded through photographs, and 19 were collected and sent to LABMEDT. In seven of the samples, it was extracted the DNA of the samples for molecular identification. DNA extraction and molecular analysis were not possible in 12 samples because of the poor conditions of the collected samples. The results of species identification, trypanosomatid infection, and the floor on which they were collected are described in Table 1, Figure 2 and Figure 3.

**TABLE 1:** Triatomine bugs captured in the residential condominium and positivity for trypanosomatids in Rio Branco, Acre, Brazil - July 2022 to July 2023.

Species	Specimen	Floor	**Positive for trypanosomatids / *T. cruzi***
*Rhodnius* sp*.* (*montenegrensis/robustus* standard)*	1	4º	NA
	2	4º	NA
	3	10º	NA
	4	10º	NA
	5	10º	NA
	6	10º	NA
Rhodnius montenegrensis**	7	4º	Positive for trypanosomatids^#^
	8	4º	Positive for trypanosomatids^#^
	9	7º	Positive for trypanosomatids^#^
	10	7º	Negative^#^
	11	7º	Negative^#^
	12	7º	Positive for trypanosomatids^#^
	13	7º	Positive for trypanosomatids^#^
	14	7º	Positive for trypanosomatids^#^
	15	7º	Positive for trypanosomatids^#^
	16	7º	Positive for trypanosomatids^#^
	17	11º	Negative^#^
	18	13º	Positive for trypanosomatids^#^
	19	2º	Positive for *T. cruzi* ^##^ (genotype TCI)
	20	2º	Positive for *T. cruzi* ^##^ (genotype TCI)
	21	2º	Positive for *T. cruzi* ^##^ (genotype TCI)
	22	2º	Positive for *T. cruzi*## (genotype TCI)
	23	2º	Positive for *T. cruzi* ^##^ (genotype TCI)
	24	2º	Negative^##^
	25	7º	Negative^##^

**Caption:** * Identification using photography. ** Identification done by morphological characteristics described by Lent and Wygodzinsky, 1979, and Rosa et al., 2012. # Analysis using optical microscopy. ## Analysis was performed using molecular biology techniques. **NA:** Specimens not analyzed were identified by photography. Meeting location: Specimens 1 and 2, Building B; 3-25, Building C.

## DISCUSSION

The occurrence of triatomine bugs in households in the Brazilian Legal Amazon has been a topic of discussion for decades^29^. However, these records have intensified in recent years, with two confirmed cases domiciled in the states of Mato Grosso and Roraima^12^ ^,^ ^30^.

Despite this, reports of the intrusion of these insects into human dwellings associated with high positivity for *T. cruzi* in specimens found are alarming, as they increase the risk of Chagas disease transmission^12^ ^,^ ^31^ ^,^ ^32^. This is because of the loss of natural ecotopes for triatomine bugs, leading them to move into residential areas attracted by artificial light or the scarcity of natural food sources^12^ ^,^ ^16^ ^,^ ^33^. 

In this study, the buildings where triatomine bugs were collected were located near forest fragments filled with palm trees, which are considered the natural habitat of triatomine bugs, especially species of the genus *Rhodnius* ^34^. Additionally, the forest fragment located near buildings may be a habitat for some mammals and a reservoir for both *T. cruzi* and *T. rangeli*, as observed in a similar study^11^. The genus *Rhodnius* is considered one of the most important genera because of the vectorial capacity of its species in the transmission of CD. In addition to its widespread distribution in the country, some species easily adapt to urbanized areas with frequent invasions into residences, being naturally infected with *T. cruzi*, such as *R. montenegrensis* ^11^ ^,^ ^35^.

*Rhodnius montenegrensis* occurs in areas with large palm trees near houses and is naturally infected by both *T. cruzi* and *T. rangeli* ^11^ ^,^ ^36^, which can be a problem because mixed infections can lead to errors in the differential diagnosis of Chagas disease^37^.

Of the seven specimens subjected to molecular analysis, five were diagnosed as positive for *T. cruzi*, all of which belonged to the TCI genotype. This Discrete Typing Unit (DTU) is widely distributed in the Americas and is frequently found in humans^3^. Its circulation is common in the Amazon region, it is present in the sylvatic cycles of the disease, and has previously been found in triatomine bugs occurring in the state of Acre^38^. Additionally, it is associated with outbreaks of Acute Chagas Disease, and is the main cause of chronic Chagas disease in Manaus, Amazonas^39^ ^,^ ^40^.

For the first time, this study described the occurrence of triatomine bugs on the 13th floor of a building. Similar studies reported the presence of this genus in residences on the 2nd and 5th floors^11^ ^,^ ^41^ ^,^ ^42^. Additionally, a study reported the occurrence of this genus on the 10th floor of a building in São Paulo, where colonies formed in the apartment where they were found^43^. All these cases share the proximity of residences to fragmented forest areas. However, the ability of these insects to reach high-rise buildings raises questions regarding the routes they take.

One hypothesis is that this locomotion may have occurred through the passive transport of objects, clothes, or reservoir animals. Ricardo-Silva et al.^30^ raised the hypothesis that pigeons could be a means of passive transport of *Triatoma maculata*, favoring its domicilization in an air conditioning unit in Roraima. Additionally, Forattini et al.^44^ have already detected this type of dispersion in *Triatoma* through birds by finding first-stage nymphs among the feathers of these animals and the colonization of these insects in their nests.

This passive dispersion is possible because some triatomines, including some species of the genus *Rhodnius*, secrete adhesive substances on their eggs, facilitating their adherence to reservoir animals such as birds^45^. This evidence suggests that reservoirs, such as birds and domestic animals, may have transported *R. montenegrensis* to the 13th floor of the building.

However, there is also the possibility of triatomines flowing directly from the palm trees to the upper floors, as species of the genus *Rhodnius* in the Amazon region are commonly found in palm tree canopies, indicating a good flying ability to move from one palm tree to another^43^ ^,^ ^46^ ^.^

Several factors may determine the induction of triatomines during flight, such as environmental (temperature) and nutritional factors^16^ ^,^ ^17^. In their experiments, Rocha et al.^17^ concluded that, as a consequence of the decrease in wild food sources due to deforestation, *R. brethesi* was led to fasting and was induced to fly two weeks later, being attracted to the nearest locations with light.

Additionally, this locomotion may have occurred floor by floor, attracted by the brightness of the upper areas of the building, as they are easily drawn to artificial light in human dwellings^46^. A study conducted in Colombia observed that triatomines of the genus *Rhodnius* were attracted by artificial light 60-110 m away from palm trees, with the peak dispersal period occurring in the early hours after dusk^47^, reinforcing the hypothesis that these insects can cover long distances, reaching the 13th floor of a building.

However, there are no studies confirming any of these hypotheses or the factors that may have induced the dispersion of *R. montenegrensis*, since each triatomine species has specific characteristics regarding flight^16^.

Nevertheless, the dispersion of triatomines in urbanized environments is concerning because this expansion increases the risk of vector-borne transmission of Chagas Disease, emphasizing the need for further studies to better understand the invasive capacity of these insects in these environments and the mechanisms involved in their locomotion process.

The occurrence of triatomines on the 13th floor of a building must be elucidated, particularly regarding the occurrence of this dispersion. Clarifying these factors is crucial for the development of new surveillance and vector control strategies.

Furthermore, considering that the collection of these insects in the condominium was conducted by residents, interventions related to human contact with insects are necessary. For example, health education projects regarding the proper management of triatomines, from prevention to collection, delivery, and surveillance, are needed. Thus, with the joint efforts of the general population and healthcare professionals, it is possible to control triatomines in urban environments.
